# Community Violence Exposure and School Functioning in Youth: Cross-Country and Gender Perspectives

**DOI:** 10.3389/fpubh.2021.692402

**Published:** 2021-07-27

**Authors:** Roman Koposov, Johan Isaksson, Robert Vermeiren, Mary Schwab-Stone, Andrew Stickley, Vladislav Ruchkin

**Affiliations:** ^1^Regional Centre for Child and Youth Mental Health and Child Welfare, Faculty of Health Sciences, UiT The Arctic University of Norway, Tromsø, Norway; ^2^Department of Epidemiology and Modern Technologies of Vaccination, Institute of Professional Education, Sechenov First Moscow State Medical University, Moscow, Russia; ^3^Child and Adolescent Psychiatry Unit, Department of Neuroscience, Uppsala University, Uppsala, Sweden; ^4^Leiden University Medical Center, Leiden, Netherlands; ^5^School of Medicine, Yale University, New Haven, CT, United States; ^6^Stockholm Center for Health and Social Change, Södertörn University, Stockholm, Sweden; ^7^Sater Forensic Psychiatric Clinic, Sater, Sweden

**Keywords:** violence, exposure, school functioning, gender, adolescents

## Abstract

**Background:** Many children and adolescents experience violent events which can be associated with negative consequences for their development, mental health, school, and social functioning. However, findings between settings and on the role of gender have been inconsistent. This study aimed to investigate cross-country and gender differences in the relationship between community violence exposure (CVE) and school functioning in a sample of youths from three countries.

**Methods:** A self-report survey was conducted among school students (12–17 years old) in Belgium (Antwerp, *N* = 4,743), Russia (Arkhangelsk, *N* = 2,823), and the US (New Haven, *N* = 4,101). Students were recruited from within classes that were randomly selected from within schools that had themselves been randomly selected (excepting New Haven, where all students were included). CVE was assessed with the Screening Survey of Exposure to Community Violence. School functioning was assessed with four measures: the Perceived Teacher Support scale, Negative Classroom Environment scale, and Academic Motivation and Perception of Safety at School scales. Multivariate Analyses of Covariance were performed to assess differences in the levels of school-related problem behaviors in boys and girls, who reported different degrees of CVE.

**Results:** Participants in all three countries reported a relatively high prevalence of violence exposure (36.2% in Belgium, 39.3% in Russia and 45.2% in the US who witnessed violence), with a higher proportion of girls than boys witnessing violent events (varied from 37.4 to 51.6% between the countries), whereas boys reported more episodes of victimization by violence than girls (varied from 32.3 to 49.9% between the countries). Youths who experienced increased CVE (from no exposure to witnessing to victimization) reported an increase in all school functioning problems in all of the countries and this association was not gender-specific.

**Conclusions:** Our findings suggest that regardless of differences in the level of CVE by country and gender, violence exposure is negatively associated with school functioning across countries. Nonetheless, even though reactions to community violence among adolescents may be expressed in a similar fashion, cross-country differences in social support systems should also be taken into account in order to provide culturally sensitive treatment modalities.

## Introduction

Community violence exposure (CVE) is usually defined as witnessing of, or victimization by a violence-related act within one's home, school, or neighborhood between individuals who are unrelated, and who may or may not know each other (1). Young populations represent a vulnerable group, with up to 1 billion children across the world being exposed to violence as both victims and witnesses annually (2). While the prevalence of CVE varies across settings and is for example, higher in the United States compared to many European countries (3, 4), the widespread nature of this phenomenon has led to it being increasingly recognized as a global public health problem (1).

Exposure to violent events can be extremely traumatic and lead to the development of severe physical, emotional or psychological problems (5). It has been linked both to clinical symptomatology and impairments in normal development, with negative consequences for mental health, substance use, social skills, relationships, and school functioning (6–10). Children with a history of violence exposure may differ from others in their developmental trajectories, often experiencing cognitive and emotional difficulties or behavioral problems, which in turn can impair their daily functioning, especially in demanding social settings, such as schools (11).

At the same time, findings regarding the impact of exposure to community violence on educational outcomes have been mixed. Although earlier studies reported no or weak relationships between community violence and academic achievement and social competence in school (12, 13), there is now growing evidence of an association between CVE and school functioning (14–17). In a nationally representative US sample of middle school students it was found that exposure to violence predicted decreased feelings of safety in school, which in turn was associated with lower student academic success (14). In another study it was similarly found that violent crime in the school neighborhood was associated with a decline in academic achievement among students (16).

In particular, it has been shown that children exposed to violence may have significant cognitive problems (e.g., attention and concentration difficulties, cognitive impairment), which negatively affect school functioning (18). Changes in cognitive function may contribute to poor performance on school assignments and tests (15). It also appears that a combination of violence exposure and subsequent symptoms of traumatic stress may create particular challenges for academic achievement (19, 20). For example, stress-related intrusive thoughts and fear of going to school may lead to increased absence from school and interfere with academic performance (15).

CVE is increasingly being evaluated in various settings and recently its cross-country aspects have also attracted attention (21–23). While some researchers postulate that the prevalence of post-traumatic stress may be consistent across different populations exposed to similar traumatic events, others argue that events considered as traumatic in the setting of one country may be differently perceived in another (24). Research assessing rates and consequences of CVE also suggests that exposure varies along several demographic characteristics and that estimates of the prevalence of CVE can differ even within a given country depending on such factors as gender and ethnicity (25, 26). Most studies report that males are more likely to encounter violent events (27), while females who experience trauma report more distress and impairment compared to traumatized males (28) and are more often diagnosed with post-traumatic stress disorder (29).

Findings on the prevalence rates of violence exposure-related trauma in Western industrialized countries have also been heterogeneous (30, 31), suggesting that different ethnic groups may be affected differently (32). African American youths more often witness and are victims of community violence than White youths (33, 34), even after controlling for demographic characteristics, such as age and gender (26). Youth exposure to community violence also varies by area of residence, with youths residing in economically disadvantaged areas (34, 35), urban areas (36), and high crime areas (37) being at greatest risk of exposure. Notably, the levels of psychosocial problems in exposed youths seem to correlate with the degree of exposure to violence (38, 39), where a greater degree of CVE (from no exposure to witnessing and to victimization) is associated with increased levels of post-traumatic stress and antisocial behavior (23, 40). Other studies have similarly indicated that in general, being directly victimized by violence is associated with a greater number of psychological problems that just witnessing violence (41). However, it remains unclear whether different levels of school functioning may similarly differ depending on the extent of CVE.

In summary, CVE is associated with a wide range of educational problems at school, but these relationships may vary by gender and between countries. The present article uses data from a large international study that collected information on CVE from culturally different samples, while also providing a gender perspective. Specifically, this study aims to: (1) assess the rates of CVE in three different countries and whether they differ by gender, and (2) explore whether a greater degree of exposure to community violence is associated with increasing problems in school-related functioning (further referred to as school functioning), whether such associations have a similar pattern in adolescents from different countries, and whether such patterns may be gender-specific. We hypothesize that boys from different countries will report higher rates of CVE than girls and that this gender difference will not be country specific. It is further hypothesized that an increasing degree of CVE will be associated with an increase in problems in school functioning, that this association may differ for boys and girls, but the patterns of these associations will nevertheless be generalizable across different countries.

## Materials and Methods

### Participants and Procedure

Data in this study were drawn from the original Social and Health Assessment (SAHA) study conducted in Belgium, Russia, and the United States (US) in 2003–2004. The primary aim of this study was to determine the factors associated with adolescent health and well-being. The study sites were the following: Belgium [the city of Antwerp (population 523,000)], Russia [the city of Arkhangelsk (population 360,000)]; and the US [the city of New Haven, Connecticut (population 125,000)]. Details of the survey and its methodology have been previously published elsewhere (42). In brief, in the Belgian and Russian locations, data were collected from a representative sample of students aged 12–18 and 12–17, respectively, in the city's public schools. The participating schools were randomly selected from a list of all schools that represented different administrative school systems and different levels of education. To obtain a representative sample of the adolescent population, a two-stage selection procedure was used, with school buildings and classes used as the units of randomization. In the US, all students aged 12–17 who were in the public school system were included. Students were recruited from within classes that were randomly selected from within schools that had themselves been randomly selected. In all countries, students completed the survey in their classrooms during a normal school day. Written informed consent was obtained from all participants prior to the survey being administered, and both parents (on behalf of their children) and children could refuse to participate. Response rates for these surveys were high with only 3.6% of children refusing to participate in Russia, <5% in Belgium and <1% in the US. For comparability, the present study was limited to adolescents aged 12–17 years old with the analytical sample thus comprising 4,743 adolescents from Belgium, 2,823 from Russia, and 4,101 from the US.

Ethical approval for the study was obtained from the Northern State Medical University in Arkhangelsk (Russia), Yale School of Medicine (US), and the University of Antwerp (Belgium).

### Measures

#### Witnessing and Victimization

Items assessing witnessed violence and violent victimization were derived from the Screening Survey of Exposure to Community Violence, developed by Richters and Martinez (43). The students were asked “about things that may happen to people in some neighborhoods.” They used a 5-point scale response format [ranging from “None” (0) to “10 or more times” (4)] to describe whether in the past year they had witnessed or been a victim of any of six types of violence (been threatened with serious physical harm, beaten up or mugged, shot or shot at with a gun, attacked or stabbed with a knife, chased by gangs or individuals, or seriously wounded in an incident of violence). Three groups were formed based on the reported degree of exposure. Those who did not witness or experience any episodes of violence were considered as the *non-exposed group*. Those who reported at least one episode of witnessing violence, but no episodes of violent victimization were considered as the *witnessing group*. Finally, those who reported at least one episode of victimization were considered the *victimization group*.

*School environment and academic motivation* were assessed with four measures. All of the measures had a similar format, with the respondents being asked to report on the truth of a number of statements using a 4-point scale (with response options ranging from definitely not true to definitely true).

Seven items were used to assess *Perceived Teacher Support* in school: (i) teachers show concern when I am absent from school; (ii) teachers are willing to help students; (iii) most of my teachers notice when I am doing a good job and let me know about it; (iv) teachers are patient when students have trouble learning; (v) teachers don't often take time to give individual attention (reversed); (vi) my teachers are unfair (reversed); (vii) I like most of my teachers this year. These items were adapted from Hawkins et al. (44), developed by the creators of the survey (42), with higher scores indicating the perception of greater teacher support. Cronbach's alpha for the scale was 0.74 for Belgium, 0.72 for Russia and 0.73 for the US.

The *Negative Classroom Environment* scale also consisted of seven items: (i) students spend a lot of class time just talking to each other; (ii) teachers spend a lot of time in class trying to get students to behave; (iii) there is a lot of fighting between students in or around the school; (iv) students don't do what the teacher has told them to do; (v) students are often late for class; (vi) students criticize or joke about teachers a lot; (vii) teachers often shout at students. These items were adapted from Kasen et al. (45), with higher scores indicating a more negative perception of the classroom environment. Cronbach's alpha for the scale was 0.71 for Belgium, 0.72 for Russia, and 0.80 for the US.

The *Academic Motivation* scale was used to assess the perceived importance of academic achievements and academic motivation. It contains six items: (i) it is important to me to get at least a B average this year; (ii) it is important to me to be considered a bright student by my teachers; (iii) it is important to me to be thought of as a good student by the other students; (iv) I try hard at school; (v) education is so important that it's worth it to put up with things I don't like; (vi) I can't wait to quit school (reversed). These items were adapted from Jessor et al. (46) and Hawkins et al. (44), with higher scores indicating increased academic motivation. Cronbach's alpha for the scale was 0.69 for Belgium, 0.67 for Russia and 0.68 for the US.

*Perception of Safety at School* was assessed with five items developed by Weissberg et al. (47), e.g., “I feel safe on the school bus or while walking to school”; “I feel safe standing in front of my school building”; and “I feel safe at after-school activities at my school.” Higher scores indicate a greater perception of safety at school. Cronbach's alpha for the scale was 0.77 for Belgium, 0.85 for Russia, and 0.82 for the US.

All of the measures used in the current study were validated in the US, including the CVE measures, and had also been used extensively in other settings internationally, including the countries included in our study (23, 48–51).

### Statistical Analyses

Data were analyzed using the Statistical Package for the Social Sciences (SPSS-23.0). Multivariate Analyses of Covariance (MANCOVA) were performed to assess differences in the levels of school-related problem behaviors in boys and girls, who reported different degrees of exposure to community violence (no exposure, witnessing, and victimization). Hence, we used a 3 (degree of CVE) × 3 (country) × 2 (gender) design for the school functioning scales. Because both exposure to violence and school functioning may vary depending on the age of the responders, all analyses were conducted while controlling for age. The level of statistical significance for the study was *p* < 0.05.

## Results

Table 1 shows the prevalence of violence exposure by gender in Belgium, Russia and the US. Students from each of the countries reported relatively high levels of exposure to community violence, with a higher proportion of girls than boys reporting witnessing violent events, whereas boys were more often victims of violence than girls (Table 1).

**Table 1:** Within-country comparisons of community violence exposure by gender [*N* (%)].

	**Belgium**		**Russia**		**US**	
	**Boys**	**Girls**	**Boys**	**Girls**	**Boys**	**Girls**
No exposure	217 (15.1)	265 (23.8)	360 (30.3)	665 (41.2)	384 (19.5)	507 (24.4)
Witnessing	503 (35.0)	416 (37.4)	443 (37.4)	663 (41.1)	763 (38.7)	1074 (51.6)
Victimization	716 (49.9)	432 (38.8)	383 (32.3)	286 (17.7)	823 (41.8)	500 (24.0)
Statistics	χ^2^ = 42.50, *p* < 0.001		χ^2^ = 87.00, *p* < 0.001		χ^2^ = 148.19, *p* < 0.001	

*χ^2^,Chi-Square (the chi-square test)*.

*p, Significance value*.

Table 2 presents the descriptive statistics (M, SD) from the MANCOVA on the perceptions of school functioning in relation to the degree of CVE for boys and girls in Belgium, Russia and the US. Compared to girls, in all of the countries boys with a similar degree of exposure generally perceived a lower level of teacher support, and reported lower academic motivation and school safety (except for in the US where girls reported lower safety at school). Subjects' scores varied on the negative classroom environment variable between the country and sex groups (Table 2).

**Table 2:** Descriptive statistics of the school environment and academic motivation [M (SD)] in Belgium, Russia, and the US by degree of community violence exposure in boys (B) and girls (G).

			**Degree of community violence exposure**		
			**Non-exposed**	**Witnessing**	**Victimization**
Teacher support	Belgium	B	22.94 (3.87)	21.59 (4.18)	20.88 (4.11)
		G	23.12 (3.38)	22.39 (3.81)	21.81 (4.28)
	Russia	B	21.78 (3.80)	21.24 (4.12)	20.22 (4.20)
		G	21.97 (3.97)	21.16 (4.13)	20.78 (4.11)
	US	B	23.51 (4.32)	22.51 (4.32)	21.56 (4.41)
		G	23.59 (4.38)	22.15 (4.48)	21.68 (4.57)
Negative classroom environment	Belgium	B	16.20 (3.84)	17.70 (3.94)	18.40 (3.87)
		G	16.06 (3.71)	17.38 (3.73)	17.76 (3.84)
	Russia	B	17.53 (4.05)	19.16 (3.64)	19.77 (3.93)
		G	18.62 (3.31)	19.31 (3.73)	19.52 (3.47)
	US	B	18.63 (4.40)	20.00 (4.28)	20.88 (4.05)
		G	19.54 (4.56)	21.00 (4.10)	21.50 (4.31)
Academic motivation	Belgium	B	15.89 (3.11)	15.40 (3.56)	15.12 (3.63)
		G	16.40 (3.30)	15.91 (3.20)	15.92 (3.25)
	Russia	B	17.55 (2.91)	17.05 (2.98)	16.34 (3.26)
		G	17.59 (2.87)	17.38 (3.11)	17.21 (2.75)
	US	B	19.62 (3.56)	19.58 (3.31)	18.67 (3.51)
		G	20.64 (2.87)	20.03 (3.01)	19.16 (3.58)
School safety	Belgium	B	17.48 (4.05)	17.00 (4.36)	16.24 (4.25)
		G	17.79 (4.06)	17.58 (3.68)	16.50 (3.95)
	Russia	B	18.54 (4.05)	18.38 (4.69)	16.97 (4.69)
		G	18.81 (4.16)	18.79 (4.13)	17.18 (4.84)
	US	B	17.94 (4.26)	17.15 (4.42)	16.06 (4.58)
		G	17.75 (4.42)	16.77 (4.35)	15.69 (4.38)

*The values presented are not adjusted for the list of covariates*.

*M (SD), Mean (Standard Deviation)*.

*US, United States*.

Table 3 presents effect sizes for each dependent variable. The main effect for the type of exposure for the total group was significant [Wilks' lambda = 0.948; *F*_(8, 18,756)_ = 63.95, *p* < 0.000, η^2^ = 0.027], with an increasingly negative perception of the classroom environment and decreasing academic motivation, perception of school safety and teacher support seen with an increasing degree of CVE (from no exposure to witnessing to direct victimization). The main effect for Gender was significant [Wilks' lambda = 0.992; *F*_(4, 9378)_ = 18.33, *p* < 0.000, η^2^ = 0.008], demonstrating a difference in the perception of the school environment with boys describing it more negatively than girls. The main effect for Country was significant [Wilks' lambda = 0.781; *F*_(8, 18,756)_ = 309.24, *p* < 0.001, η^2^ = 0.117], suggesting differences in the school variables between students in Belgium, Russia and the US. Students from the US had the highest negative perception of the classroom environment, academic motivation and teacher support and lowest school safety; students from Russia had the highest school safety and lowest teacher support scores; students from Belgium reported the least negative perception of the classroom environment and academic motivation. Finally, the main effect for Age was also significant [Wilks' lambda = 0.960; *F*_(4, 9,378)_ = 96.65, *p* < 0.001, η^2^ = 0.040], with an increasingly negative perception of the classroom environment, decreasing academic motivation, poorer perception of school safety and teacher support with increasing age. The interaction effect for the Degree of CVE × Country was weakly significant [Wilks' lambda = 0.996; *F*_(16, 28,650)_ = 2.39, *p* < 0.01, η^2^ = 0.001], indicating some differences in school functioning in relation to the degree of violence exposure between the countries. Specifically, while students from the US had a more negative perception of the classroom environment which increased from no exposure to victimization, students from Russia reported higher school safety which decreased from no exposure to victimization. The tests of between-subjects effects showed that there were weak differences related to the perception of school safety and negative classroom environment, whereas for the other school variables there were similar patterns between the countries in relation to an increasing degree of CVE. The interaction effect for Gender × Country was significant [Wilks' lambda = 0.995; *F*_(8, 18,756)_ = 5.78, *p* < 0.001, η^2^ = 0.002], suggesting that gender differences in the perception of the school variables followed different patterns in different countries. Compared to boys, girls in the US had a more negative perception of the classroom environment, higher academic motivation and lower teacher support and school safety; girls in Russia had a more negative perception of the classroom environment and higher academic motivation, teacher support, and school safety; girls in Belgium had a less negative perception of the classroom environment and higher academic motivation, teacher support, and school safety. The follow up between-subjects tests showed that these gender differences were significant for all of the school variables except academic motivation. The interaction effect for the Degree of CVE × Gender was significant [Wilks' lambda = 0.995; *F*_(8, 18,756)_ = 2.04, *p* < 0.05, η^2^ = 0.001]. The follow up between-subjects tests indicated that the significant effect was related to differences in the perception of the classroom environment with boys having a more negative perception in relation to increasing CVE than girls. Finally, the interaction effect for the Degree of CVE × Country × Gender [Wilks' lambda = 0.998; *F*_(16, 28,650)_ = 1.30, ns, η^2^ = 0.001] was not significant, indicating that the gender-specific patterns of school variables occurring in response to varying degrees of CVE were similar in all of the countries.

**Table 3:** Effect sizes for each dependent variable and summary statistics (η^2^, p).

	**Teacher support**	**Negative classroom environment**	**Academic motivation**	**School safety**
Age	0.004, <0.001	0.010, <0.001	0.022, <0.001	0.004, <0.001
Violence exposure	0.018, <0.001	0.031, <0.001	0.008, <0.001	0.021, <0.001
Country	0.010, <0.001	0.062, <0.001	0.144, <0.001	0.009, <0.001
Gender	0.001, <0.01	0.001, <0.01	0.006, <0.001	0.000, ns
Violence exposure × Country	0.001, ns	0.002, <0.01	0.001, ns	0.001, <0.05
Violence exposure × Gender	0.000, ns	0.001, <0.01	0.000, ns	0.000, ns
Gender × Country	001, <0.05	0.004, <0.001	0.000, ns	0.001, <0.01
Violence exposure × Country × Gender	0.000, ns	0.001, ns	0.001, ns	0.000, ns

*η^2^, Eta-squared statistic*.

*p, Significance value*.

*ns, Non-significant*.

As the differences by CVE, country, and gender could have been masked as a result of simultaneously assessing all of the outcomes in one model, we further examined associations for each of the outcomes individually using UNIANCOVAs, in order to determine whether the patterns would hold. The results were essentially the same.

## Discussion

The purpose of this cross-sectional study was to assess the rates of CVE in different countries and whether they differ by gender. We also aimed to explore whether an increasing degree of CVE in adolescents from different countries is associated with greater problems in different areas of school functioning and whether there may be certain patterns of associations, including gender-specific patterns that may be generalizable across different countries.

The overall findings showed that adolescents from each of the three countries reported a relatively high prevalence of exposure to community violence and that girls more often reported witnessing violence, while boys more often reported being victims of community violence. The proportion of youth in the current study who witnessed violence (36.2% in Belgium, 39.3% in Russia, and 45.2% in the US) was higher than reported in other studies. For example, according to a 2011 US victimization survey, 36.4% of 14 to 17-year-old adolescents witnessed an assault in their community during the previous year (52). In another large-size study of 9 to 18-year-old youths from seven European countries, 34% reported experiencing at least one incident of community violence exposure during the past year (53). Our study results thus further underline that youth exposure to community violence is a global public health issue that warrants increased attention and action to protect the most vulnerable population groups.

The present cross-country study also sought to explore the relationship between an increasing degree of violence exposure and school functioning from country and gender perspectives. Overall, youths who experienced increased CVE (from no exposure to witnessing to victimization) reported an increase in a broad range of problems related to school functioning. This finding partly accords with the results of an earlier study that examined the relationship between exposure to community violence and academic functioning in a small sample of economically disadvantaged, African-American middle-school students, which found that exposure to community violence had only a weak relationship with academic functioning in general, but that the relationship was intensified under certain circumstances (54). Specifically, children who had very high achievement expectations and a very strong moral-religious emphasis were most at risk for poor academic functioning as exposure to community violence increased (54).

We also found that with a similar degree of exposure, boys (as compared to girls) in all of the countries generally perceived a lower level of teacher support, and reported lower academic motivation and school safety (except for in the US), while results were more mixed between country and gender groups for perceptions of a negative classroom environment. Our results suggest that while there may be country- and gender-specific differences in school functioning, and that increased problems in relation to an increasing degree of violence exposure may be gender-specific (with more problems related to school functioning among boys), the pattern of this gender-specific association was generalizable across different countries. From this perspective, these findings appear congruent with the notion found in the child trauma literature that there is a “universality of trauma responses” (55, 56). Accumulating evidence worldwide indicates that violence exposure can chronically and pervasively impact multiple developmental domains, including social, biological, psychological, and cognitive functioning, including academic outcomes (57, 58). Considering that failure in school can have long-term detrimental consequences for competency and social adjustment as adolescents transition into adulthood (59, 60), measures aimed at eradicating or reducing community violence may be beneficial in order to increase youth adjustment and ensure better social functioning in the future.

An extended discussion of the mechanisms linking CVE and school functioning is beyond the scope of the current paper although different factors might be involved. Some of the reported differences in the association between CVE and the perceived school environment may be related to an underlying comorbid psychopathology. In particular, previous research (11, 61) has shown that adolescents exposed to CVE may develop a number of mental health problems, which may impact on their school functioning. For example, depressed adolescents may perceive their teachers as being less supportive and have less academic motivation in general (50), while also perceiving the classroom environment as being more negative because of their decreased mood and a pessimistic perspective on life (62). Similarly, it is also possible that children with anxiety or post-traumatic stress symptoms/disorder (possibly resulting from community violence) may perceive their school environment as being less safe (63) as well as describe student–teacher relationships as poorer (64). Alternatively, it is possible that delinquency might play a role in this association. Specifically, earlier research has linked CVE to childhood delinquency (65). In terms of the current study this might be important as phenomena such as aggressive behavior and engaging in bullying have been, respectively, linked to lower levels of teacher support (66), and school safety (67), while a recent article has reported a relationship between problematic behaviors (disciplinary infractions) in school and a negative perception of the school environment (68). As yet, the exact factors linking CVE and delinquency are uncertain although some of the suggested potential mechanisms such as depression and anxiety (69) might also be important for school functioning as outlined above, which highlights the importance of prospective research to determine exactly how CVE is linked to school functioning and whether the relationship is direct or mediated by other variables.

Similar cross-country, and in some respects, gender non-specific dynamics in school functioning in relation to the extent of community violence suggest that reactions to community violence among adolescents may be expressed in a similar detrimental fashion. Given this, the results of this study suggest that efforts aimed at reducing CVE may be important when it comes to improving school adjustment in adolescents. Decreasing the stress related to exposure to violence may potentially improve school engagement and academic motivation. Having said this, additional research is also warranted as before the notion that there are culturally invariant responses to trauma can be accepted, cross-country differences in social support systems should also be considered. Indeed, an enhanced understanding of CVE and the factors associated with it will further enable culturally sensitive treatment modalities to be provided if and where necessary (70).

The main strength of the current study includes the use of data from large adolescent community samples from three different countries with different ethnic backgrounds and varying levels of violence exposure. This study also has several limitations that should be mentioned. First, the use of a cross-sectional design prevented us from determining causality. Second, we relied on adolescent self-reports of CVE which may have been affected by different types of reporting bias e.g., social desirability bias. However prior studies have indicated that self-reports of behavior by adolescents tend to be valid and reliable (71, 72). Third, by including heterogeneous countries like the US, Belgium, and Russia together in the study, we cannot be certain that the CVE-academic functioning associations we observed are not due to other unmeasured variables, such as e.g., those associated with differences in race/ethnicity which were not considered in the study. At the same time, finding similar patterns of associations in all three countries, in spite of potentially unmeasured factors, suggest that the results were not country specific. Finally, reports on exposure to community violence were limited to the last year and therefore we were not able to identify children with longer histories of exposure to community violence.

## Conclusion

In spite of the substantial differences in the rates of CVE between different countries, we found that girls more often witnessed violence, whereas boys tended to more often experience violent victimization. Our results further suggest that adolescents exposed to community violence may be at risk for a variety of problems in relation to school functioning and these associations seem to have a similar pattern in different countries, irrespective of gender. The results of this study suggest that efforts to reduce CVE may be important when it comes to improving school adjustment in adolescents.

## Data Availability Statement

The datasets used in this article are not readily available because the data cannot be shared publicly due to the initial decision of the local ethical committees, as well as the restrictions included in the informed consent statement (where it was stated that the data would only be used by the research group and would not be transferred elsewhere). Requests to access the datasets should be directed to Roman Koposov, roman.koposov@uit.no.

## Ethics Statement

The studies involving human participants were reviewed and approved by the Ethics committee at the Northern State Medical University in Arkhangelsk (Russia), the Ethics committee at the Yale School of Medicine (US), and the Committee for Medical Ethics at the University of Antwerp in Belgium. Written informed consent to participate in this study was provided by the participants' legal guardian/next of kin.

## Author Contributions

VR, RK, JI, RV, MS-S, and AS were involved in the conceptualization and design of the study. RK and VR conducted the data collection and drafted the manuscript. VR conducted the data analysis. All authors reviewed and edited the manuscript.

## Conflict of Interest

The authors declare that the research was conducted in the absence of any commercial or financial relationships that could be construed as a potential conflict of interest.

## Publisher's Note

All claims expressed in this article are solely those of the authors and do not necessarily represent those of their affiliated organizations, or those of the publisher, the editors and the reviewers. Any product that may be evaluated in this article, or claim that may be made by its manufacturer, is not guaranteed or endorsed by the publisher.
